# Determine the therapeutic role of radiotherapy in administrative data: a data mining approach

**DOI:** 10.1186/1471-2288-15-11

**Published:** 2015-02-03

**Authors:** Jina Zhang-Salomons, Greg Salomons

**Affiliations:** Division of Cancer Care and Epidemiology, Queen’s Cancer Research Institute, Queen’s University, Kingston, ON Canada; Department of Oncology, Queen’s University, Kingston, ON Canada; Department of Public Health Sciences, Queen’s University, Kingston, ON Canada; Department of Physics, Queen’s University, Kingston, ON Canada; Kingston General Hospital, Kingston, ON Canada

**Keywords:** Role of radiotherapy, Treatment intent, Palliation, Administrative data, Missing data, Data quality, Classification, Decision tree

## Abstract

**Background:**

Clinical data gathered for administrative purposes often lack sufficient information to separate the records of radiotherapy given for palliation from those given for cure. An absence, incompleteness, or inaccuracy of such information could hinder or bias the study of the utilization and outcome of radiotherapy. This study has three specific purposes: 1) develop a method to determine the therapeutic role of radiotherapy (TRR); 2) assess the accuracy of the method; 3) report the quality of the information on treatment “intent” recorded in the clinical data in Ontario, Canada. A general purpose is to use this study as a prototype to demonstrate and test a method to assess the quality of administrative data.

**Methods:**

This is a population based retrospective study. A random sample was drawn from the treatment records with “intent” assigned in treating hospitals. A decision tree is grown using treatment parameters as predictors and “intent” as outcome variable to classify the treatments into curative or palliative. The tree classifier was applied to the entire dataset, and the classification results were compared with those identified by “intent”. A manual audit was conducted to assess the accuracy of the classification.

**Results:**

The following parameters predicted the TRR, from the strongest to the weakest: radiation dose per fraction, treated body-region, disease site, and time of treatment. When applied to the records of treatments given between 1990 and 2008 in Ontario, Canada, the classification rules correctly classified 96.1% of the records. The quality of the “intent” variable was as follows: 77.5% correctly classified, 3.7% misclassified, and 18.8% did not have an “intent” assigned.

**Conclusions:**

The classification rules derived in this study can be used to determine the TRR when such information is unavailable, incomplete, or inaccurate in administrative data. The study demonstrates that data mining approach can be used to effectively assess and improve the quality of large administrative datasets.

## Background

Radiotherapy is an important type of treatment for cancer. The role of radiotherapy in the management of cancer could be either for curing the disease or for palliation when the disease is deemed incurable. Along with the differences in the anticipated clinical outcomes, radiotherapy is prescribed differently in curative and palliative settings [1]. Many clinical studies concerning radiotherapy require the knowledge of the therapeutic role of radiotherapy (TRR).

There has been an increased interest in studying the utilization of palliative radiotherapy at the population level [2–7]. These studies require the researchers to correctly separate the treatments given for palliation from those given for cure. If the information on the TRR is incomplete in the data, the utilization rates could be seriously underestimated. On the other hand, if the goal of the study is to investigate the outcome of the curative radiotherapy, an incorrect classification of the TRR (for example, many palliative treatments were misclassified as curative treatments) could lead the researcher to observe an outcome poorer than the actual outcome.

Notwithstanding its importance, there has not been a consistent and reliable way to determine the TRR. Most population-based studies rely on administrative data, in which treatment “intent” is recorded by radiation oncologists and entered into their computer system by a radiotherapist or clinical clerk. However, such information is not always available [2] and transcription errors could occur. Moreover, treatment “intent” could change during the course of treatment, depending on the response to treatment and the progression of the disease. For these reasons, researchers often apply certain rules to enhance the information on “intent”. These rules were primarily based on the number of fractions given in a course of treatment combined with other information such as body-region and time-to-death to distinguish palliative from curative treatments [4, 8].

There are challenges in adopting these rules to determine the TRR. For example, in order to establish a cutoff for the number of fractions given in a palliative course of treatment versus a curative course, we first need to group the consecutive treatments into courses. Many clinical systems used to capture treatment information do not combine the records of treatment into courses. In the cases where the treatments are combined into courses, the definition of course varies. A shorter survival time after treatment often indicates that a patient has received palliative therapy. However, vital statistics are not readily available in clinical databases. In addition, the establishment of these rules are arbitrary in nature, and the validity of these rules has not been assessed.

We developed a data driven approach that utilizes the treatment parameters captured in the Record and Verify System (R&V system) during the treatment planning and delivery to determine the TRR. Using a decision tree method, we derived a set of classification rules and applied these rules to the clinical data in Ontario, Canada. We also conducted a manual audit to evaluate the validity of these rules. The method described here can be applied to other data sets or variables to assess and improve the quality of administrative data.

## Method

**Figure 1:**
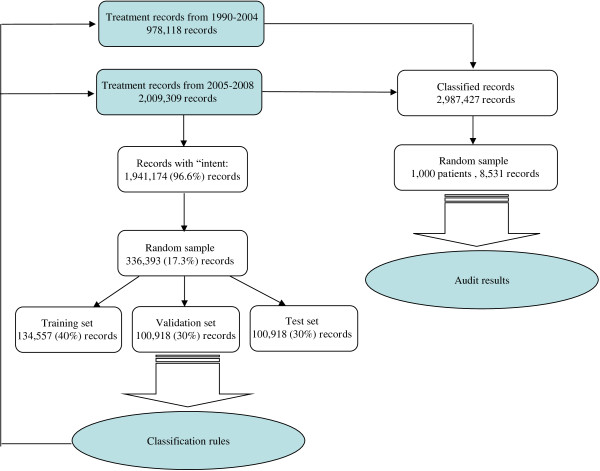
**The treatment records used in the induction and validation of classification rules.**

1. **Source of data**
  Before April 2004, radiotherapy in Ontario was provided by eight Regional Cancer Centres managed by a provincial agency Cancer Care Ontario (CCO). All clinical activities were managed through the Oncology Patient Information System (OPIS). After an episode of treatments were given, treatment details on paper records were summarized and entered into the OPIS by a radiotherapist. Variables in the radiotherapy summary data include start and end date of treatment, total dose (in cGy), total number of fractions, all irradiated body-regions, treatment techniques, and treatment “intent”.
  Since 2005, the radiotherapy clinics in Ontario were integrated into their hosting hospitals. The radiotherapy data were collected by each hospital and submitted to CCO in a standard data format, the CCO Databook format [9]. These data were extracted from the R&V System by treating hospitals. There are two common R&V Systems in North American: Aria (Varian Medical Systems, Palo Alto, CA) and Mosaiq (Elekta, Stockholm, Sweden). Both systems record similar information on treatment activities and have the ability to archive the data to an external storage. Both systems are used in Ontario. In the R&V System, each daily activity of radiotherapy is recorded as a separate record. The information transferred to CCO includes registration date, treatment date, irradiated body-region, fraction-size (dose per fraction), treatment technique, and treatment “intent”. These data can be readily linked to hospital’s central patient system to obtain information on disease site and stage at diagnosis.
  For the purpose of this study, we have linked the clinical data to the Ontario Cancer Registry to isolate the records of treatment given to malignant cancers. We also attached the date and cause of death to the radiotherapy data for the purpose of audit. The use of the data for this study is approved by the Health Sciences Research Ethics Board of Queen’s University (Study ID: ONGY-134-00, Ethics Romeo file# 6005177).
2. **Rule induction**
  We used the data on the treatments given between April 2005 and November 2008, collected in the CCO Databook format, to derive the classification rules. The study data set contained 336,393 treatment records randomly selected from the records with “intent” assigned in the treating hospitals. Overall, 14% of the treatment records were for palliation, affecting 34% of the patients. The analysis was conducted using SAS Enterprise Miner software (SAS Institute, Cary, North Carolina, USA).
  Disease sites (ICD codes) were grouped into site groups according to anatomic region and the likelihood of being treated with radiotherapy. Irradiated body-regions were grouped into body-region groups according to proximity and to reflect whether a body-region was likely to be treated palliatively or curatively. Treatment dates were expressed as the number of days elapsed since the patient started the first treatment for the same cancer. Radiotherapy techniques were classified to identify single-field versus multi-field treatments. The above variables were used as classification variables. Treatment “intent” was grouped into a binary (curative versus palliative) outcome variable “intent-flag”.
  We used decision tree analysis with Chi-square Automatic Interaction Detection (CHAID) algorithm [10] to grow the classification tree. The initial “tree trunk” was all treatment records in the study dataset. The data set was split into two groups based on the value of each classification variables to see if there is a statistically significant difference in the value of classification variable between the two groups. The most significant split was used to form the first branching of the tree. The same process was repeated for each of the new groups formed, until a further split would lead to a group with less than 400 records. The final branches of the tree contained a series of groups that were maximally different from one another on intent-flag. At each step, a chi-square test with Bonferroni adjustment was performed to determine whether a significant split could be made. Only the splits that achieved a p-value less than 0.2 were retained. English classification rules were derived by following the path from the root to each tree branch.
3. **Missing data**
  In the study data set, all variables except treatment date had missing values. The classification rules derived using all variables would not apply when one of the classification variables had a missing value. We therefore grew 7 different trees using subsets of classification variables. These 7 trees were ranked by their misclassification rate; the model with lowest misclassification rate received highest ranking. When the full model could not be applied to a record due to missing values in one or more classification variables, models with less classification variables were used. When more than one model could be used in classification, the model with highest ranking was used.
4. **Validation**
  **Internal validation**
  The study data set was randomly divided into three subsets for training (40%), validation (30%), and testing (30%). The training set was used to grow the tree; the validation set was used to calculate the misclassification rate; the test set was used to grow a second tree to assess the reliability.
  **Manual audit**In the internal validation described above, the accuracy of each classification tree was calculated by assuming that the intent-flag represents the true TRR. In reality, this is not always the case due to human errors in data entry and changes in treatment protocols. A disagreement between intent-flag and the TRR derived from the classification rules could reflect errors in the data instead of an incorrect classification. To assess the true error rates of classification, we conducted a manual audit using the treatment records of 1,000 randomly selected patients. The random sample was stratified by treatment centre, treatment period (before or after April 2005), and whether or not the classified TRR agreed with the intent-flag. All treatments ever received by these patients were pulled from the database. The records were further linked to other data sources to obtain the stage at diagnosis and the vital status of patients to assist the identification of the “true” TRR. The final audit records were reviewed by an experienced medical physicist to classify the treatment into palliative or curative. The audit is blinded, in which the medical physicist did not have access to the intent-flag nor the derived TRR. The data sets used in rule induction and validation are illustrated in Figure 1.

## Results

1. **Profile of study variables**
  Table 1 shows the profile of the variables used in the final tree classifier. Treatment technique did not contribute to the final model, and therefore was not shown in the table. The result shows that the average fraction-size for the treatments with a curative “intent” was smaller than that for the treatments with a palliative “intent”. The average number of days elapsed between a given fraction and the first fraction was more for curative than for palliative treatments. The total number of curative treatments versus palliative treatments varied by treated body-region and disease site. Column 4 shows that all variables except treatment date had missing values in the data set. Overall, 85% of the total treatment records had complete information on all four classification variables.

**Table 1:** **Classification variables used in decision tree analysis**

Classification variables	Description		Completeness ^*^
	Curative	Palliative	
Fraction size (cGy)	Median: 200	Median: 300	99%
Time from the first treatment (no. of days)	Median: 19	Median: 9	100%
Body-region group			91%
Chest	123,378 (91%)	12144 (9%)	
Organs and tissues in pelvis region	78,220 (97%)	2211 (3%)	
Pelvis – single side	1,057 (44%)	1332 (56%)	
Pelvis – both sides	27,902 (91%)	2685 (9%)	
Brain	9,177 (52%)	8589 (48%)	
Neck	16,378 (92%)	1336 (8%)	
Head	14,256 (93%)	1020 (7%)	
Bone – spine, limb, chest, head	3,817 (21%)	14353 (79%)	
Abdomen	7,206 (73%)	2614 (27%)	
Skin	983 (73%)	357 (27%)	
Other regions	7,116 (96%)	262 (4%)	
Disease site group			97%
Head/neck(140–144,146-149,160,161)	26,090 (97%)	924 (3%)	
Other head/neck	3,735 (92%)	309 (8%)	
Colon/intestines (152,153)	1,782 (58%)	1,271 (42%)	
Rectum (154)	17,585 (91%)	1,709 (9%)	
Liver (155)	140 (38%)	226 (62%)	
Other GI (150,151,156-159)	6,736 (74%)	2,349 (26%)	
Lung (162–165)	18,850 (55%)	1,5319 (45%)	
Bone (170)	245 (68%)	114 (32%)	
Soft tissue (171)	1,942 (86%)	303 (14%)	
Melanoma (172)	1,390 (54%)	1,204 (46%)	
skin (173)	134(85%)	23(15%)	
Breast (174,175)	97,728 (93%)	6,897 (7%)	
Ovary (183)	461 (46%)	541 (54%)	
Other GYN (179–182,184)	1,2791 (90%)	1,391 (10%)	
Prostate/Testis/Penis (185–187)	74,061 (95%)	3,896 (5%)	
Bladder (188)	2,206 (67%)	1,075 (33%)	
Kidney (189)	464 (24%)	1,504 (76%)	
CNS (190–192)	7,524 (93%)	604 (7%)	
Thyroid/Endo (193,194)	727 (70%)	318 (30%)	
Unspecified group 1 (195,196)	1,271 (88%)	176 (12%)	
Unspecified group 2 (197–199)	4,152 (66%)	2,153 (34%)	
Lymphoid/leukemia(200,202,204-208)	7,295 (71%)	2,933 (29%)	
Hodgkin’s disease (201)	1,697 (94%)	116 (6%)	
Myeloma (203)	484 (24%)	1,548 (76%)	

*The completeness was calculated using all records of treatment given between 2005 and 2008; while the summary statistics were based on the records used in decision tree analysis, which are randomly selected from the records without missing data.

**Figure 2:**
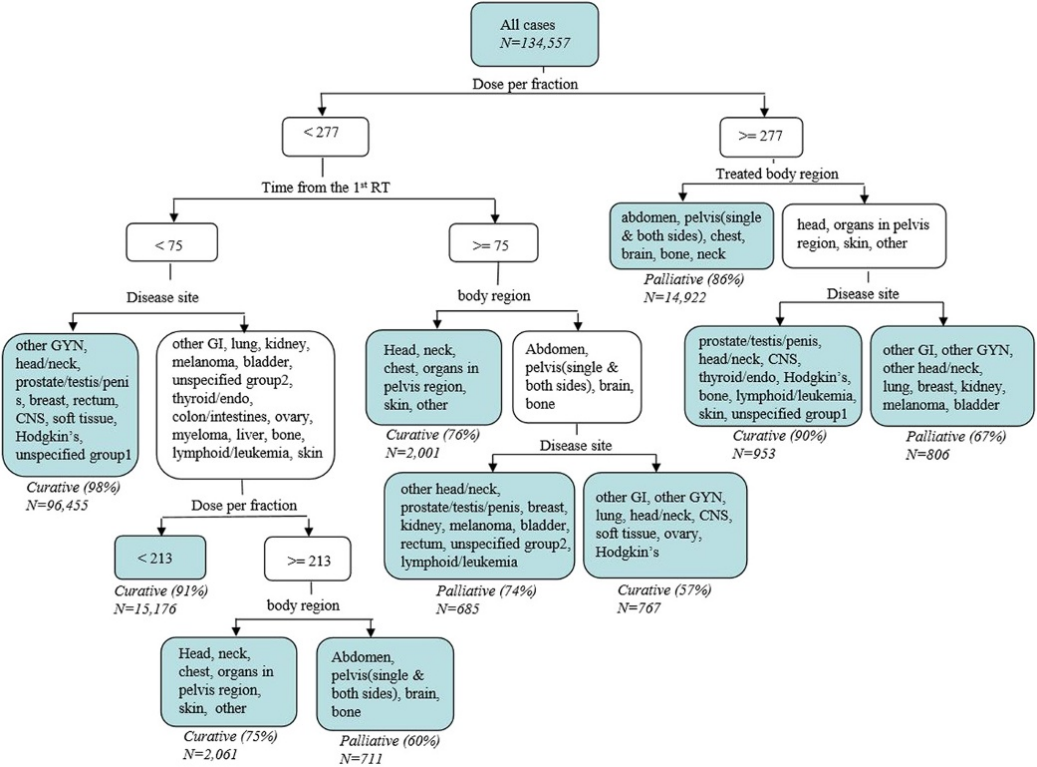
**The classification tree that predicts the therapeutic role of radiotherapy using treatment parameters.**

2. **Classification tree**The classification tree generated using all four classification variables is shown in Figure 2. The training data set was first divided up into two groups according to fraction-size. The records with a fraction-size greater or equal to 277cGy were further divided into two groups according to body-region. The first group contains treatments given to abdomen, chest, brain, bone, neck, and pelvis. The majority of the records in this group had a palliative “intent”. The treatments given to other body-regions were further divided up into two groups according to disease site. The majority of the records in the first group had a curative “intent”, which include the prostate/testis cancer patients received radiotherapy to organs in pelvis region; head and neck cancer patients received radiotherapy to head, skin cancer patients received radiotherapy to skin. The other tree branches could be interpreted in the same fashion. The branches lead to a total of 10 end points, each were classified either as curative or palliative according to the “intent” of the majority records in that subgroup.

The above tree structure was further described as a set of English rules by following the path way that leads to each of the end points. We also developed 7 different sub-trees using only 3, 2, or 1 classification variables. The sub-trees and the classification rules are available from the authors upon request.

3. **Internal validation**

Shown in Table 2 were the accuracy of the 7 different classification trees, calculated by internal validation. The table shows that, model 1 (the full model) performed best on the validation data set. Among the “misclassified” records, 2.2% were classified as palliative while the intent-flag was curative, and 3.3% were classified as curative while the intent-flag was palliative. Model 2 (removing disease site) had a very similar accuracy as model 1, while it “misclassified” slightly more palliative records into curative. Model 3 (removing body-region) had a slightly lower accuracy than model 1 and 2. Model 4 (removing both body-region and disease site) had a very similar accuracy as model 3. However, model 4 “misclassified” more palliative treatments into curative than model 3 did. Removing fraction-size from the full model (model 5, 6 and 7) resulted in a marked decrease in the performance of the models, suggesting that fraction-size was the strongest predictor.

**Table 2:** **Model accuracy assessed by internal validation for 7 different models**

Model	Classification variables	Number of rules	Accuracy (CI)	Confusion matrix	
Model 1	Fraction-size	10	94.6% (94.4%, 94.7%)	C to P*:	2.2%
	Disease site			P to C:	3.3%
	Body-region				
	Time from the 1^st^ treatment				
Model 2	Fraction-size	13	94.5% (94.3%, 94.6%)	C to P:	1.9%
	Body-region			P to C:	3.6%
	Time from the 1^st^ treatment				
Model 3	Fraction-size	10	94.2% (94.0%, 94.3%)	C to P:	2.4%
	Disease site			P to C:	3.4%
	Time from the 1^st^ treatment				
Model 4	Fraction-size	8	94.1% (94.0%, 94.3%)	C to P:	2.0%
	Time from the 1^st^ treatment			P to C:	3.8%
Model 5	Disease site	18	92.0% (91.8%, 92.2%)	C to P:	3.0%
	Body-region			P to C:	4.9%
	Time from the 1^st^ treatment				
Model 6	Body-region	11	91.0% (90.8%, 91.2%)	C to P:	2.4%
	Time from the 1^st^ treatment			P to C:	6.6%
Model 7	Disease site	10	90.1% (89.9%, 90.3%)	C to P:	3.8%
	Time from the 1^st^ treatment			P to C:	6.2%

*’C’ refers to curative, ‘P’ refers to palliative.

The tree structure remained the same after repeating the analysis using the test dataset, except for minor changes in the percentage of curative/palliative cases at the endpoints. This second tree yields the same classification rules and therefore is not shown.

4. **Manual audit**

Table 3 shows the performance of the classification rules evaluated through an audit in which manual classifications were used as “gold standard”. In this analysis, sensitivity, specificity, positive and negative predictive values were calculated using palliation as the target outcome. Among the records for which the classification rules and the intent-flag yielded the same classification (sample A), the classifiers performed very well, with sensitivity, specificity, positive and negative predict values all above 95%. When the classification rules and the intent-flag disagreed (sample B), the classification rules did better in identifying curative treatments (specificity 66.0%) while the intent-flag did better in identifying palliative treatments (sensitivity 36.3%). Overall, the classification rules were more effective in identifying curative treatments than identifying palliative treatments. If a treatment were given for palliation, it would be classified as palliative in 84.2% of the times. If a treatment were given for cure, it would be classified as curative in 98.5% of the times. The predictive power of the classification rules was high for both curative and palliative treatments. If a record were classified as palliative, the likelihood of being truly palliative was 92.3%; If a record were classified as curative, the likelihood of being truly curative was 96.8%.

**Table 3:** **Validity of the classification rules based on a manual audit of the treatment records of 1,000 randomly selected patients**

	Sample A*	Sample B**	Entire sample***
Sensitivity (palliative)	95.5 (94.2,96.8)	36.3 (34.0,38.5)	84.2 (82.9,85.5)
Specificity (curative)	99.5 (99.4,99.7)	66.0 (63.4,68.5)	98.5 (98.3,98.7)
Positive predictive value (palliative)	97.3 (96.3,98.3)	58.6 (55.8,61.5)	92.3 (91.3,93.3)
Negative predictive value (curative)	99.2 (99.0,99.4)	43.8 (41.6,45.9)	96.8 (96.5,97.0)

*From the records for which the classification rules and the intent-flag agreed.

**From the records for which the classification rules and the intent-flag disagreed.

***Adjusted for sampling stratification.

Utilizing the audit results, we estimated the misclassification rates for the entire data set (column 2 and 3 of Table 4). For the treatments given between 2005 and 2008, 3.4% records did not have an intent-flag, 4.2% was misclassified by the classification rules and 4.1% was misclassified by the intent-flag. The proportion of records correctly classified was 95.8% by the classification rules and 92.5% by intent-flag. The classification rules performed fairly well when applied to the treatments given before 2005, which were collected in a different format. For the entire study period between 1990 and 2008, 96.1% of the records were correctly classified by the classification rules. Due to the poor availability of information on “intent” in the earlier period, only 77.5% of the records were successfully classified by intent-flag. Auditing only the records for which the classification rules and the intent-flag disagreed over-estimated the success rates by approximately 1% (column 4 and 5).

**Table 4:** **The performance of the classification rules and the intent-flag for the records of treatments given between 1990 and 2008 in Ontario**

2005-2008				
	Entire sample*		Sample B only**	
	Classification rules	Intent-flag	Classification rules	Intent-flag
	(95% CI)	(95% CI)	(95% CI)	(95% CI)
% correct	95.8 (95.5, 96.1)	92.5 (92.2, 92.7)	96.8 (96.6, 96.9)	93.4 (93.2, 93.5)
% incorrect	4.2 (3.9, 4.4)	4.1 (3.9, 4.4)	3.2 (3.1, 3.4)	3.2 (3.1, 3.4)
% missing	0	3.4***	0	3.4
**1990-2008**				
	**Entire sample**		**Sample B only**	
	**Classification rules**	**Intent-flag**	**Classification rules**	**Intent-flag**
	**(95% CI)**	**(95% CI)**	**(95% CI)**	**(95% CI)**
% correct	96.1 (95.8, 96.3)	77.5 (77.2, 77.7)	97.1 (96.9, 97.2)	78.3 (78.1, 78.4)
% incorrect	3.9 (3.7, 4.2)	3.7 (3.5, 4.0)	2.9 (2.8, 3.1)	2.9 (2.8, 3.1)
% missing	0	18.8	0	18.8

*Estimated through the audit of a random sample drawn from all treatment records.

**Estimated through the audit of a random sample drawn from the records for which the classification rules and the intent-flag disagreed.

***CI was not provided because the percentage of records missing intent was observed instead of being estimated through the audit.

## Discussion

This study demonstrates that the TRR could be adequately predicted by treatment parameters such as fraction-size, body-region, treatment time line, and disease site. This finding is important because the knowledge of TRR is needed for population-based clinical studies, especially in the studies concerning palliative radiotherapy. Since treatment “intent” is not an essential parameter for the delivery of radiotherapy, not all clinics routinely collect information on “intent”. When the information on “intent” is not available, the classification rules described here could be used as a reasonable tool to impute the data.

The decision tree presented here can also be used as a tool to aid the quality assessment of administrative data. Using administrative data to conduct clinical research has become a well established methodology among health care communities. However, the credibility of these studies depends, to a great extent, on the quality of data [11]. Because the collection of administrative data is often not monitored and controlled, human errors in data entry are inevitable. The traditional approach of data quality assessment requires retrospective review of medical records by trained medical staff, which is labor intensive. Given the massive volume of administrative data, such an audit is often not viable. In this study, we isolated the records whose intent-flag did not follow the prevailing trend displayed by the majority of records for audit purpose. This targeted approach, although it slightly underestimated the error rate, makes a routine audit of administrative data much more feasible.

Other predictive models such as logistic regression could also be used in this setting. We chose to use a decision tree because it has a higher interpretability than a regression model [12]. This facilitates a clinical validation and provides opportunity for manual pruning of a specific branch. In the classification tree shown in Figure 2, the various branches can be explained by basic principles of radiation oncology. For example, the predominance of fraction-size as a differentiator between curative and palliative treatments comes from the basic radiobiological principles of cell survival and repair that guide radiation oncology. Since palliation is not so concerned with the late effects of radiation, it does not require the small fraction-sizes necessary for curative treatments [13]. Despite this basic principle, there are some disease sites such as prostate where hypo-fractionated regimens are being promoted [14]. This can be seen on the right hand side of Figure 2, where more accurate classification was obtained when the fraction-size information was combined with disease information. For the disease sites such as prostate and skin, treatments with a high fraction-size were still considered curative, although this accounted for less than 1% of the treatment fractions given.

For the treatment records with smaller fraction size (left hand side of Figure 2), the next determinant factor was the time interval between a given treatment and the time when a patient received his/her first treatment. From Figure 2, it appeared that the treatments given more than two-and-a-half months after a patient started his/her first treatment were more likely to be palliative compared with the treatments given within the first two-and-a-half months. This reflects the fact that re-treatments are most likely to be palliative. One may question why the median value of time-from-the-first-treatment for curative treatments (19) was larger than the same median value for palliative treatments (9, Table 1). These median values reflect the fact that curative treatments have a longer course than palliative treatments.

In evaluating the classification rules, we have calculated the sensitivity and specificity of the classification. However, one needs to keep in mind that classifying the TRR of treatments is different from identifying a case of disease. Both curative and palliative cases were treated with radiation, except with different total dose and fraction size. On occasions, the boundary of the classification is not very clear. One example would be when a prescribed curative course of treatments was terminated early due to various reasons. One could argue that these treatments remain as curative because they were prescribed for cure. We have encountered such cases during the manual audit, when a typical course of curative treatments was terminated and switched to palliation to other body regions with a different fraction size, or a patient died of a heart failure during a course of curative treatments. We have classified these treatments as “attempted cure” in the audit. Because our model uses dose per fraction instead of total dose for a course, these treatments would be classified as curative by the classification rules.

This study has several limitations. First, the information included in decision tree is limited primarily to treatment parameters. The stage of disease, co-morbidity, patient’s performance status, the prior and concurrent treatment and supportive care which a patient received, and a short survival time are all potential predictors of TRR. Unfortunately, such information is not available through the R & V system; obtaining such information requires linking radiotherapy data to other data sources. Although the linked data could potentially improve the accuracy of the classification, it increases the difficulty of the application of the classification rules, particularly in the jurisdictions where linkage of clinical data to other data sources is not a routine practice. The performance status of cancer patients are not routinely collected at the cancer clinics in Ontario. A routine collection of such data would strengthen population-based clinical research.

Second, the performance of the classification rules depends on the quality of the classification variables. The fraction-size and treatment time are automatically entered into the R&V system at the time of treatment, and therefore are an accurate recording of how much treatment a patient received and when. The irradiated body-regions are also recorded at the time of treatment. However, body-regions are often recorded as verbatim descriptions, and the interpretation of such information is challenging for computerized analysis. In Ontario, the body-region descriptions are manually coded into standard body-region codes; human errors (interpretation, transcription, typographical) inevitably occur during the coding process. Likewise, primary disease site recorded in hospitals are subject to the same types of human errors. Because the decision tree model describes the general pattern in the data, these errors are not likely to affect the model itself. However, if the classification variables of an individual record is erroneous, the record could be misclassified. This, perhaps, has in part contributed to the fact that body-region and disease site did not predict the therapeutic role as well as fraction-size did.

Third, the classification rules are built upon the existing overall pattern of radiotherapy practice. The performance of the tree classifier depends on the uniformity and consistency of radiotherapy practice. If current practice changes, the rules will need to be updated to reflect these changes. For example, there is currently a trend towards hypo-fractionated treatments such as stereotactic beam radiotherapy for small cell lung tumors. If these practices become standard care, the classification tree may need to rely more heavily on body-region and disease site information. In addition, diseases with a very low incidence rate that do not follow the general pattern of treatment may not be captured correctly. Therefore, applying this classification rules to the treatments given to rare disease might be problematic.

Finally, the applicability of the derived classification rules in other jurisdictions remains to be tested. Because all radiation treatment units use R&V systems to plan and deliver treatments, the data used in this study should be available to all radiotherapy clinics worldwide. On the other hand, Although the biological basis guiding the prescription of radiotherapy is the same, minor variations in practice across geographic regions may exist. Our audit results showed that the performance of the tree classifier varied from hospital to hospital in Ontario (data not shown). Although this could reflect the variation in data quality, it could also in part reflect the minor differences in clinical practice in different hospitals. For the best results, separate classification trees should be derived using regional or institutional data to verify the validity of the classification rules.

## Conclusions

The classification rules derived in this study can be used to determine the TRR when such information is unavailable, incomplete, or inaccurate in administrative data. The study demonstrates that data mining approach can be used to effectively assess and improve the quality of large administrative datasets.
